# Do alternative reproductive tactics facilitate evolutionary rescue? A comment on Knell & Parrett 2024

**DOI:** 10.1093/evlett/qraf049

**Published:** 2025-12-31

**Authors:** Jana M Riederer, Franz J Weissing

**Affiliations:** University of Groningen, Groningen Institute for Evolutionary Life Sciences, Groningen, the Netherlands; University of Groningen, Groningen Institute for Evolutionary Life Sciences, Groningen, the Netherlands

**Keywords:** extinction, reproductive strategies, sexual selection, model

## Abstract

This contribution is a comment on a simulation study of Robert J. Knell and Jonathan M. Parrett (Evo. Lett. 8, 539–349, 2024). There is growing evidence that in ecological adaptation, sexual selection is a “double-edged sword”—it can fuel adaptation and population persistence, or hinder adaptation and lead to population extinction. Knell and Parrett explore this topic, using an individual-based model to investigate how alternative reproductive tactics (ARTs) affect adaptation to changing environments. They find that in the presence of ARTs, extinction can be averted, as fixed ARTs facilitate evolutionary rescue. While we appreciate their research question and approach, we question the generality of this result. First, some of their conclusions hinge on the parameter values chosen. In their model, individuals express one of two reproductive tactics (fighting or sneaking) depending on whether their condition exceeds a given threshold. We demonstrate that their conclusions rely strongly on this threshold value. For high values (i.e., when most individuals sneak), they observe evolutionary rescue—however, for values lower than those explored in K&P, fixed ARTs do not impact extinction. When we allow the threshold to evolve, it evolves to take low values, indicating that when fixed ARTs are adaptive, they do not promote evolutionary rescue. Second, K&P assume that the sneaking strategy results in considerably lower mating success than the fighting strategy. We show that, if the average mating success of the sneaking strategy is increased, fixed ARTs again do not cause evolutionary rescue. Finally, we show that varying the degree of influx of variation or the inheritance process similarly breaks the association between fixed ARTs and evolutionary rescue. Overall, we agree with K&P that ARTs may influence evolutionary rescue, but possibly in different contexts than those considered in their manuscript. Thus, whether and how ARTs shape extinction risk remains an open question.

## Introduction

The impact of sexual selection on ecological adaptation and population extinction dynamics has long been a topic of interest (e.g., Candolin & Heuschele, 2008; Fricke & Arnqvist, 2007; Godwin et al., 2020; Reding et al., 2013). There is growing evidence that sexual selection can both facilitate and hinder adaptation to environmental change, as well as both prevent and fuel population extinction. Knell & Parrett (2024) address this question in the context of alternative reproductive tactics (ARTs), utilizing individual-based simulations to investigate whether and how the presence of ARTs affects adaptation and population persistence in a changing environment. While we appreciate their research question and approach, we are not entirely convinced by the generality of their results, as they hinge on parameter values that are not evolutionarily stable and assumptions that may not be biologically plausible.

Their model considers a population in a changing environment, where individual condition is proportional to the degree of adaptation to the current environment. Depending on their condition, males adopt one of two mating tactics. Males in good condition develop into “major males” that engage in contests for matings, whereas males in poor condition develop into “minor males” that sneak matings. These condition-dependent (and, hence, plastic) ARTs can be either “fixed” (i.e., minor males can only sneak) or “simultaneous” (minor males can both sneak and compete in contests). Here, we focus on the model variant with fixed ARTs, which produces the more spectacular and counterintuitive results, and these results also form the focus of K&P 2024. In this model variant, the mating success of major males is contingent on their condition and thus on their degree of adaptation (as males in better condition are more likely to win the mating competition), whereas the mating success of minor males is unrelated to their condition (for more details, see Supplementary Material 1). In this setup, K&P proposed that sexual selection reinforces natural selection and thus accelerates adaptation—but only if the best-adapted males have higher reproductive success. In contrast, ARTs bypass this by allowing males in poorer condition to gain matings through “sneaking” tactics. Given this, they predict that sexual selection can better facilitate evolutionary rescue in the absence of ARTs than in their presence. In other words, in systems with ARTs, extinction should be more common. Contrary to their expectations, their simulations show that extinction is less common in the presence of fixed ARTs (while simultaneous ARTs accelerate extinction, see also Supplementary Material 3). The authors explain this by positing that in the presence of fixed ARTs, top-quality males face fewer competitors (as lower-quality males do not participate in mating contests but rather sneak matings), which increases the reproductive success of top-quality males and thus their contribution to the gene pool of future generations.

Individual-based simulations are a valuable tool for exploring questions in evolutionary theory, and especially in the context of evolutionary rescue, for a variety of reasons. They incorporate the effects of stochasticity and individual variation more easily than most analytical approaches, allow fitness to emerge from the model, and (perhaps most importantly) force us to be explicit about the details of the proposed mechanisms (Kuijper et al., 2012; Long & Weissing, 2023). However, individual-based simulations also necessitate careful interpretation, as they can be highly idiosyncratic (not unlike empirical systems), and by necessity the model findings rely on parameter values and model assumptions, the consequences of which are not always readily apparent. We here want to highlight two sets of assumptions in K&P 2024, which demonstrate that their conclusions are not as general as the authors suggest, namely assumptions regarding the mating system and assumptions regarding the inheritance and mutation process.

## Assumptions regarding the mating system

**Figure 1.**
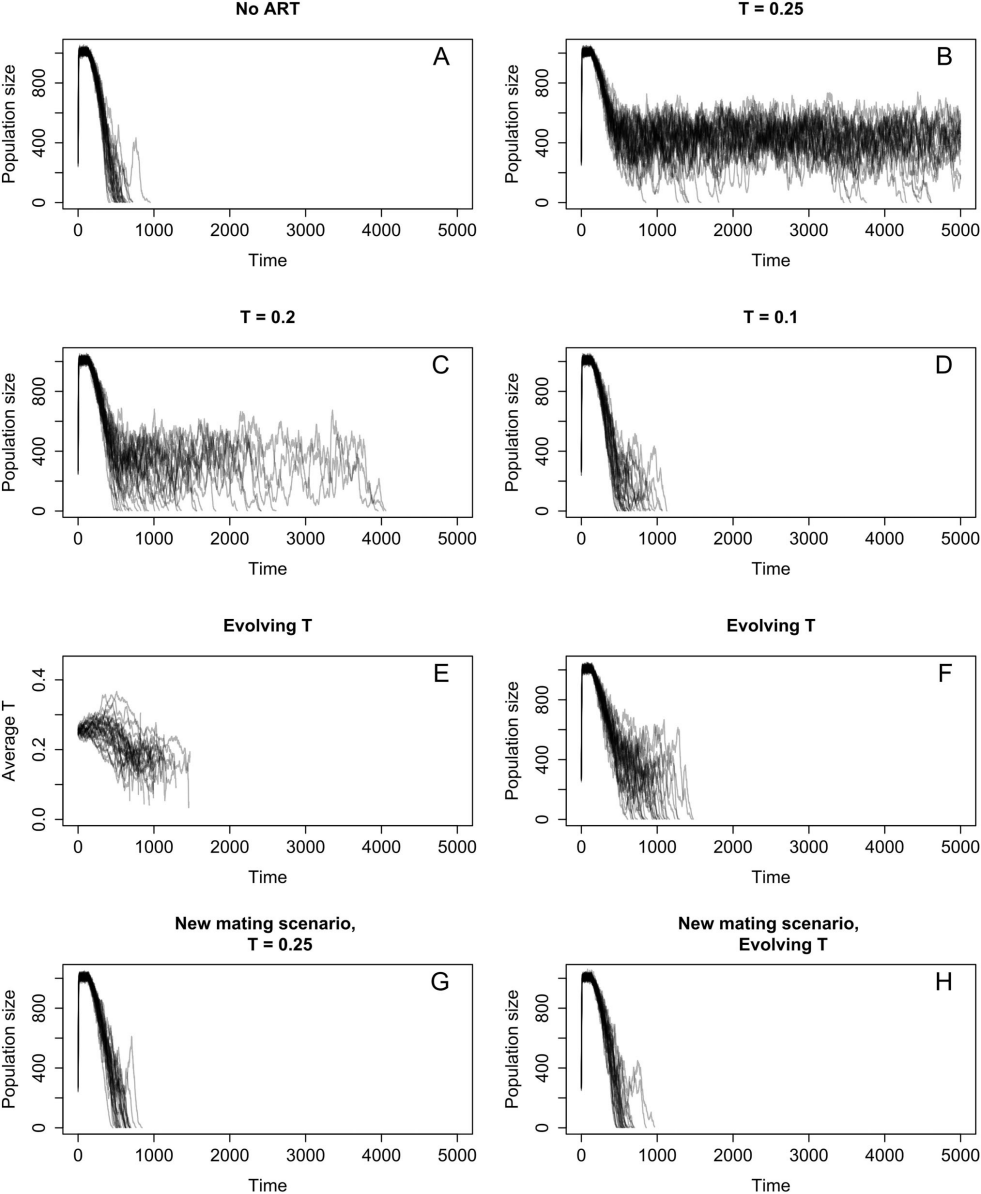
Dependence of evolutionary rescue on the presence of alternative reproductive tactics (ARTs) and the threshold *T*, the level of individual condition at which males switch mating tactics. Each panel depicts 30 replicate simulations. **(A, B) Simulations for the parameter settings considered by K&P in their Figure 1**. (A) In the absence of ARTs, population extinction occurs in all 30 replicate simulations. (B) In the presence of ARTs, evolutionary rescue ensues in most of the 30 replicate simulations. **(C, D) Evolutionary rescue no longer occurs in the presence of ARTs if K&P’s default threshold value (*T* = 0.25) is reduced**. (C) A slight reduction to *T* = 0.20 leads to population extinction in all 30 simulations, albeit on a somewhat longer timescale than considered by K&P. (K&P consider 500 timesteps, corresponding to about 60 generations, all simulations shown here were run for 5000 timesteps.) (D) A larger reduction to *T* = 0.10 leads to population extinction within 500 to 1000 timesteps. **(E, F) Evolutionary instability of the threshold value *T* = 0.25**. (E) When the threshold is initially set at *T* = 0.25 but can subsequently evolve, *T* evolves to take on lower values. (F) Consequently, ARTs no longer prevent population extinction. **(G, H) Effect of a change in the mating system**. If the mating system is changed in such a way that females have a higher tendency to mate with sneaker males when major males are in short supply, ARTs do not prevent population extinction, neither in the case of a fixed threshold value (*T* = 0.25, panel G) nor in the case of an evolving threshold (panel H).

In K&P’s model, males adopt a mating strategy depending on whether their condition (or “quality”) is above or below a certain threshold. As the authors rightly point out, the results depend on the value one assumes for this threshold. The higher the threshold, the more efficient evolutionary rescue is, as more males in poor condition are “filtered” from the pool of competitors. Figure 1 demonstrates that this principle extends to threshold values below those shown in K&P 2024. Specifically, if the threshold is sufficiently low, no evolutionary rescue occurs (Figure 1C, D). It is thus crucial to consider what values we should reasonably expect the threshold to take: which parts of parameter space should we expect to reflect natural conditions? One way to answer this question is to allow the threshold itself to evolve, rather than fixing it at a predetermined value (for details, see Supplementary Material 2). Interestingly, we see that if the threshold is the result of adaptive evolution, ARTs never prevent extinction (Figure 1F), as thresholds consistently evolve to be low (Figure 1E). And this is not surprising, since adopting the sneaking tactic is only adaptive if it results in reproductive success equal to or higher than the one this individual could have obtained through competing in a contest. Therefore, we expect that males should evolve to sneak matings only when this increases their reproductive success—at which point the ART no longer “filters” individuals from the pool of competitors that would have stood to gain from competing. In other words, K&P state that ARTs facilitate evolutionary rescue because in the presence of ARTs, low-condition males “exclude themselves from the contests for females,” allowing “males with the best, most adapted genes to father even more offspring than they would do otherwise.” However, this can only be true if adopting an alternative tactic reduces the reproductive success of minor males and is thus maladaptive.

It is important to note at this point that other aspects of the mating system shape what threshold values are selected. K&P assume that the sneaking strategy is, by and large, highly unsuccessful compared to engaging in contests for matings, leading to a selection pressure for low threshold values and low mating success for minor males. This is indeed the case in some empirical systems with plastic ARTs (e.g., Hunt & Simmons, 2001); however, it need not always be. For instance, in various empirical systems with ARTs, it depends on environmental conditions which tactic is more advantageous, often resulting in (on average) similar or equal reproductive success for the two tactics (e.g., Michalczyk et al., 2018; Schradin & Lindholm, 2011; Shah & Rubenstein, 2022; reviewed in Brockmann & Taborsky, 2008). Specifically, K&P assume that every female mates with a major male, but only a fraction of females mate with minor males, thus putting the minor strategy at an inherent disadvantage (for details, see Supplementary Material 1). If we change this assumption, increasing the average success of the sneaking tactic (by allowing females to mate with multiple sneaker males when major males are rare; for details, see Supplementary Material 2), we see that ARTs again do not prevent extinction—this time, both when the threshold is free to evolve (Figure 1H) and also when the threshold is fixed at high values (such as the values used in K&P 2024, Figure 1G). This can be easily understood by considering that the sneaking strategy is no longer by default associated with low reproductive success and can thus no longer serve to “filter out” lower-quality males. Overall, we can thus conclude that fixed ARTs only prevent extinction if the minor strategy is both highly unsuccessful (compared to the major strategy) and employed in a maladaptive way (i.e., maladaptively employed by males in reasonably good condition).

Of course, in nature, we can expect that mating strategies may at times be maladaptive. For instance, rapid environmental change may be associated with an evolutionary lag, which could result in a maladaptively high (or low) threshold. Under such circumstances, the results of K&P—that ARTs facilitate evolutionary rescue—may indeed hold (if the threshold is maladaptively high), or conversely, ARTs may induce even more rapid extinction (if the threshold is maladaptively low). Thus, while a maladaptively high threshold is not biologically impossible, it is an important assumption to acknowledge and keep in mind.

## Assumptions regarding inheritance and the mutation process

**Figure 2.**
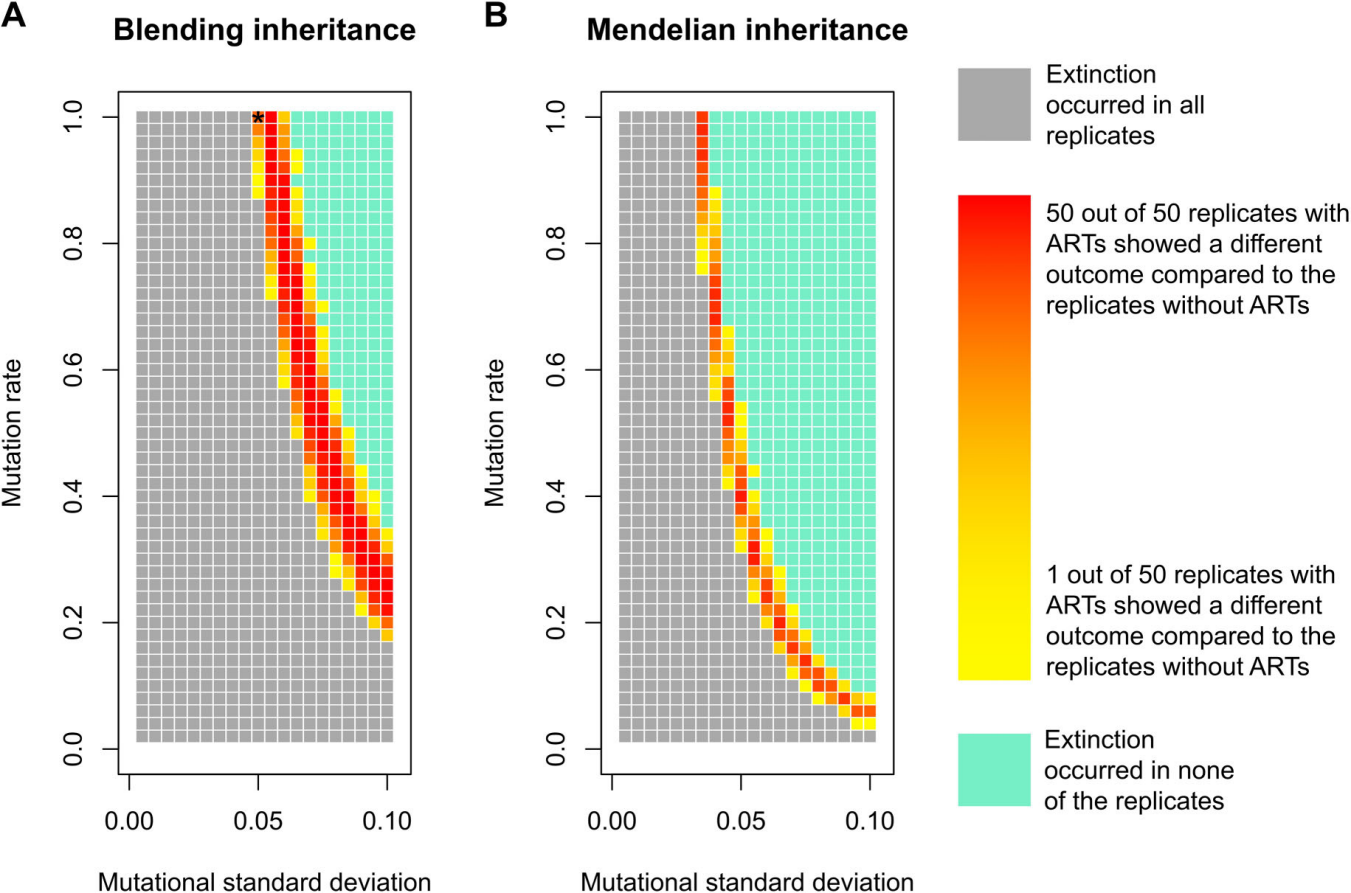
Extinction risk under different levels of mutational variability, for **(A) the blending inheritance scenario** considered by K&P and **(B) a Mendelian inheritance scenario** (see also Supplementary Material 2). For various combinations of mutation rate and mutational standard deviation, each small square represents 100 replicate populations: 50 replicates with ARTs (*T* = 0.25) and 50 replicates without ARTs (*T* = 0). The color of a square is based on the number of replicate populations that went extinct within 5,000 timesteps. Grey: all replicates went extinct. Turquoise: no replicates went extinct. Yellow to red: there is a difference in the extinction rate between simulations with and without ARTs; the closer to red, the bigger the difference. K&P consider a mutation rate of 1.0 and a mutational standard deviation of 0.05, the corresponding square is indicated in the left panel with an asterisk. All other parameter settings are the same as those in K&P 2024, Figure 1. ARTs impact the extinction rate within a limited window. If the mutational variability is too low (corresponding to a low mutation rate and/or a low mutational standard deviation) extinction always occurs. If the mutational variability is too high (corresponding to a high mutation rate and/or a high mutational standard deviation), extinction never occurs. Under which combinations of the mutation rate and the mutational standard deviation ARTs impact extinction, depends on whether we, like K&P, assume blending inheritance (Figure 2A) or Mendelian inheritance (Figure 2B).

A second set of assumptions, particularly important in the context of evolutionary rescue, concerns the generation of variation. For evolutionary rescue to occur, evolution must keep pace with environmental change, which depends on the availability of sufficient genetic variation. Testing different levels of mutational variability (determined by the mutation rate and the standard deviation of the distribution of mutational step sizes) reveals that even when the threshold for adopting the major tactic is maladaptively high (i.e., in the scenario considered by K&P), ARTs only affect the outcome within a limited window (Figure 2A). Specifically, if the influx of variation is too low, population extinction always ensues (irrespective of the presence or absence of ARTs). Conversely, if the influx of variation is too high, extinction never ensues (irrespective of the presence or absence of ARTs). Where exactly this window lies further depends on other factors such as the inheritance system. As explained in the Supplement, the assumptions of K&P’s model correspond to blending inheritance. If this is changed to Mendelian inheritance with diploid individuals, the “goldilocks” zone in which ARTs can have an impact shifts substantially, both quantitatively and qualitatively (Figure 2B).

## Conclusions

In summary, in the model considered in K&P 2024, ARTs only prevent extinction and facilitate evolutionary rescue under very specific conditions: the sneaking strategy must yield low reproductive success but must nevertheless be widely (and maladaptively) employed, and the mutational variability of the trait adapting to the changing environment must fall within a narrow window.

However, whilst we argue that in the model presented by K&P, ARTs are unlikely to facilitate adaptation and prevent extinction, they may do so in other contexts. For instance, it may be that environmental change exacts a greater viability cost in major males, which may engage in physical contests when competing for matings. In this case, the option of a second, less energetically costly mating strategy may allow for increased male survival, improving population viability and buffering against extinction, “buying time” for evolutionary rescue to occur. Moreover, ARTs allow males to express a broader range of phenotypic variation, exposing a greater part of morphospace to natural selection. This may speed up evolution and allow some of this variation to become co-opted into ecological adaptation. Similarly, in many systems with ARTs, the mating strategies and their associated morphological, physiological, and behavioral traits are plastically expressed—previous studies have shown that such plasticity may enhance evolvability, e.g., by shaping the distribution of genetic mutations or by allowing the build-up of cryptic genetic variation (Brun-Usan et al., 2021; Feiner et al., 2021; van Gestel & Weissing, 2016). Finally, it is worth noting that, as mentioned in K&P 2024, this model considers a type of ART in which the sneaking tactic is making the “best of a bad job,” that is, only low-quality males, who are in too poor a condition to compete, sneak. This is not always the case—in many mating systems with ARTs, the different tactics are not condition-dependent (e.g., Alonzo & Sinervo, 2001; Zipple et al., 2024), with potentially vastly different impacts on evolutionary rescue. Thus, as also emphasized in K&P 2024, ARTs are diverse and cannot easily be captured in a single model, and depending on the details of the system, the outcome may vary considerably.

Over the past two decades, there has been considerable debate on the interplay between sexual selection, adaptation, and extinction. Both empirical and theoretical studies provide evidence for sexual selection either facilitating adaptation and evolutionary rescue (Cally et al., 2019; Fricke & Arnqvist, 2007; Godwin et al., 2020; Lorch et al., 2003; Lumley et al., 2015) or for sexual selection hindering adaptation and leading to extinction (e.g., Baldauf et al., 2014; Chenoweth et al., 2015; Doherty et al., 2003; Reding et al., 2013). K&P 2024, as well as our present comment, add to this literature demonstrating that the interplay of sexual selection and ecological adaptation is complex, and that the specific properties of a biological system can significantly impact this interplay.

## Supplementary Material

qraf049_Supplemental_File

## Data Availability

All data and code underlying the results are available on GitHub, at https://github.com/Jana17/AlternativeReproductiveTactics_Comment_2025/.
