# Supplementary material for: Do alternative reproductive tactics facilitate evolutionary rescue? A comment on Knell & Parrett 2024
Source: Evol Lett. 2025 Dec 31;10(2):147–51. doi: 10.1093/evlett/qraf049 (PMC13043900; doi:10.1093/evlett/qraf049)
Supplement: qraf049_Supplemental_File [file qraf049_supplemental_file.pdf]

Do alternative reproductive tactics facilitate evolutionary rescue? A comment on Knell & Parrett 2024: supplementary material

Jana M. Riederer & Franz J. Weissing

University of Groningen, Groningen Institute for Evolutionary Life Sciences, Nijenborgh 7, Groningen, the Netherlands

Email addresses: j.m.riederer@rug.nl and f.j.weissing@rug.nl

Contents

1. The model in Knell & Parrett 2024

2. Additions and changes to the K&P model

3. Replication of Figure 1 in Knell & Parrett 2024

4. References

1. The model in Knell & Parrett 2024

Knell and Parrett (henceforth K&P) simulate both "simultaneous" and "fixed" alternative reproductive tactics (ARTs), as well as both directional and stepwise environmental change. As we focus on the scenario with fixed ARTs and directional environmental change, this summary of their model does not include information on the other scenarios.

Model overview

K&P consider a population of males and females, with overlapping generations, living in a changing environment. Over the course of the simulation, these individuals can mate, reproduce, and die. Each individual has a heritable phenotype that determines the degree of adaptation to the current environment and influences individual condition. Depending on their condition, males use one of two alternative mating strategies: they either compete for matings ("major males") or try to sneak matings ("minor males"). Since the trait that adapts individuals to their environment is heritable (with mutations), it evolves over time. However, if adaptation does not keep pace with the changing environment, the population may decline and eventually go extinct. The question is whether, when and how the presence of ARTs affects the survival prospects of the population. K&P's simulation code is written in R.

Environmental change

The environment E_t at time *t* is characterised by a single continuous variable. All individuals in the population experience the same environment. During the first 125 timesteps, E_t fluctuates around the value 1: $E_t = 1 + \delta E$, where δE is drawn from a uniform distribution between -0.02 and +0.02. After 125 timesteps, E_t undergoes directional change: $E_{t+1} = E_t + \delta E$, where δE is drawn from a normal distribution with mean 0.005 and standard deviation 0.005.

Individual characteristics

Individuals live for several timesteps and thus experience different environmental states. Each individual has an age that is zero at birth and increases by one with each timestep. Age affects maturation and the survival probability from one timestep to the next. An individual (whether male or female) is considered mature at age three (according to the *R* code; the article suggests that the age of maturity could be two).

Each individual *i* is characterised by an "environmental genotype" G_i and a specified amount of resources R_i, which is determined at birth and is constant throughout the individual's lifetime. The match between G_i and the environment E_t at time *t* determines the (genetic) degree of adaptation of the individual at that time. The resource level R_i is determined stochastically and is drawn from a uniform distribution. The individual's resource level and its adaptation to the current environment determine the "condition" $C_i(t)$ of individual *i* at time *t*: $C_i(t) = R_i - |G_i - E_t|$. Individuals with $C_i(t) < 0$ die. Individual condition affects fecundity in females and the mating tactics of males. Males, whose condition at age one is above a threshold *T*, develop into "major" males, while the other males develop into "minor" males. The threshold *T* is externally given, fixed throughout the simulation and the same for all individuals. Upon sexual maturation, major males develop a "display trait" *D* (i.e., some form of sexual ornament or armament) that, for simplicity, is equal to a male's condition. This

display trait is thus given by $D_i(t) = C_i(t)$ for major male *i*. The display trait carries a viability cost. Females, minor males, and juvenile major $D_i(t)$ males do not express a display trait.

Individual survival

Individual survival depends on age, the size of the individual's display trait (for sexually mature major males), and population density. According to the article (K&P 2024), the probability $P_i(t)$ that individual *i* dies at the end of timestep *t* is given by the equation:

$$P_i(t) = 0.46 + 0.169 \cdot A_i(t) + 0.0154 \cdot A_i(t)^2 + D_i(t) \cdot c + N_t / K \,, \qquad (1)$$

where $A_i(t)$ is the individual's age at time *t*, $D_i(t)$ is the size of the individual's display trait, *c* are the costs per unit display trait (default value: $c = 2$), and N_t / K is the quotient of the population size N_t at time *t* and the carrying capacity *K*. However, equation (1) cannot be correct, as irrespective of $A_i(t)$ and $D_i(t)$, it yields a death probability exceeding one for all individuals (and, hence, immediate population extinction) if $N_t / K > 0.54$. This does not align with Figure 1 in K&P 2024, where population sizes are close to the carrying capacity for hundreds of timesteps. Moreover, irrespective of population size N_t, the equation yields a death probability exceeding one for all mature individuals (i.e., all individuals with $A_i(t) \geq 3$).

The simulation code in the Appendix of K&P 2024 indicates that K&P used a different equation:

$$P_i(t) = 0.08 \cdot \left(0.46 - 0.169 \cdot A_i(t) + 0.0154 \cdot A_i(t)^2 + D_i(t) \cdot c + N_t / K\right). \qquad (2)$$

In our comment, we used equation (2) for all simulations. As, according to equation (2), $P_i(t)$ is not bounded between zero and one, we set all death probabilities above one to one and all negative probabilities to zero. Note that, since $D_i(t) = C_i(t)$ for major males, the survival of major males declines with individual condition. In other words, major males that are better adapted to their current environment have a lower survival probability.

Mate choice – major males

In each time step, mature females can mate with mature major males and mature minor males. The competition among major males for matings is implemented as follows. All mature major males are randomly assigned to "mating groups" of *g* individuals (default value: *g* = 6), and the males within each group compete for matings. (All groups are of size *g* – if the number of major males cannot be divided by *g* without remainder, random major males are allocated to the last group until it has reached size *g.* These males are thus present in more than one group.) Within each group, the *g* major males are ranked according to the size of their display ornament *D,* the male with the largest ornament is top-ranked. Each (mature) female is assigned at random to one of the mating groups and mates with a male within that group. Since there are usually more females than groups of males, most groups are assigned to several females. Females prefer to mate with the highest-ranked male in a group. Specifically, the chosen male is drawn from the mating group using a weighted lottery where the

probability of major male *i* being chosen is proportional to $rank_i^{-\beta}$. Hence, for the default value, the probability that the 6th-ranked male in the group is chosen by a given female is a factor $\frac{1}{36}$ smaller than the probability that the top-ranked male is chosen. The parameter β can be interpreted as the strength of female preference or the impact of rank on winning a contest. Note that the mating prospects of major males only depend on the rank of their display traits and not on the display traits themselves. Minute differences in condition can therefore have a substantial effect on the mating probabilities.

Mate choice – minor males

In addition to mating with a major male, each female can also mate with a minor male. The probability of a female mating with a minor male is given by:

$$S(t) = \frac{N_{minor}(t)}{N_{minor}(t) + N_{major}(t)} \cdot S_{\max}. \quad (3)$$

Here, the quotient denotes the relative frequency of mature minor males in the mature male population, while $S_{\max}$ is the maximal probability that a female mates with a minor male. If the female does mate with a minor male, a random mature minor male is chosen for reproduction. The default setting for $S_{\max}$ is $S_{\max} = 0.5$. Hence, the probability that a given female mates with a minor male is between zero and 0.5, whereas the probability of the female mating with a major male is one.

Reproduction

The fecundity of females depends on their condition. The litter size of mated female *i* at time *t* is $F_i(t) = C_i(t) \cdot k$, rounded to the nearest integer (we use the value of *k = 6*, as stated in the caption of K&P 2024 Fig. 1). Moreover, reproduction is density dependent (this is only specified in the *R* code and not in the Methods section of K&P 2024), as the probability of each of the $F_i(t)$ offspring to be actually born is $1 - N_t/K$ (and zero if the population size N_t is above the carrying capacity *K*). If the female has mated with multiple males (i.e., with a major male and a minor male), then for each offspring, paternity is assigned at random to one of the potential fathers (with equal probability).

Inheritance

The "environmental genotype" G_i of an offspring *i* with parents *m* and *n* is given by the average $\frac{1}{2}(G_m + G_n)$ of the two parental genotypes plus a mutation value drawn from a normal distribution with mean zero and standard deviation 0.05. This inheritance system resembles "blending inheritance", which is incompatible with Mendelian inheritance (e.g., Avise 2014). In the case of blending inheritance, all offspring of a pair of parents have the same genotype (in the absence of mutation), while Mendel's laws of segregation and recombination lead to considerable variation among offspring. In our report, we used K&P's inheritance system as the standard setting but also considered a Mendelian variant of the K&P model.

2. Additions and changes to the K&P model

We re-implemented the code of K&P 2024 in C++ and explored several changes to the setup considered by K&P. The simulations in K&P 2024 were run for 500 timesteps, which corresponds roughly to 50-60 generations. However, within this runtime, equilibrium was often not yet achieved, and the simulation outcome remained unclear. We therefore allowed our simulations to run for 5000 timesteps (i.e., roughly 500 to 600 generations). Within this timespan, most replicates either reached equilibrium or went extinct. Descriptions of the other changes are given below.

Implementation of an evolving threshold

In K&P 2024, males develop into minor or major males depending on whether their condition C is below or above a threshold T. This threshold is implemented "globally," i.e., it remains constant over time and is the same for all individuals in the population. We additionally explore a model version where this threshold represents an "individual strategy" that can evolve. In this model, each individual *i* has a heritable phenotypic trait T_i that determines the threshold used by that individual. It is inherited in the same manner as the environmental genotype G_i, that is, the T_i-value of an offspring individual is equal to the average of the two parental values, plus a mutation value drawn from a normal distribution with mean zero and standard deviation 0.05. As the threshold is bounded between zero and one, mutations that would result in $T_i < 0$ or $T_i > 1$ are redrawn until $0 \le T_i \le 1$.

Implementation of an alternative mating system

In K&P 2024, all mature females mate with a major male, but only a fraction $S(t) \le S_{\max} = 0.5$ of the females mates with a minor male, even if the minor males vastly outnumber the major males. We additionally explore an alternative implementation of the mating system, in which the expected number of minor males that a female mates with is not given by $S(t)$ as specified in eqn (3), but by:

$$E(t) = \frac{N_{minor}(t)}{N_{major}(t)} \cdot E_1, \quad (4)$$

where E_1 denotes the expected number of matings with minor males in the case that the quotient in (4) is equal to one, that is, when mature minor and major males are equally abundant. With our default value $E_1 = 0.5$, each minor male is still at a disadvantage with respect to the major males, but if the minor males vastly outnumber the major males, females can mate with more minor than major males. Specifically, we assume that, for any given female, the number of matings with minor males is drawn from a Poisson distribution with expected value $E(t)$. Obviously, eqn (4) is not well-defined for $N_{major}(t) = 0$. If no major males compete for matings (i.e., $N_{major}(t) = 0$), we assume (in line with K&P 2024) that all females mate with one (randomly chosen) minor male.

Implementation of Mendelian inheritance

As the inheritance system in K&P 2024 is not biologically realistic, we also explore a scenario that considers Mendelian inheritance. To this end, each individual is endowed with a gene locus that harbours alleles determining adaptation to the current environment. Individuals are diploid, and an individual carrying alleles a_i and a_j develops a phenotype $G_{ij} = \frac{1}{2}(a_i + a_j)$ that determines adaptation to the environment in the same way as the "environmental genotype" does in the K&P model. In particular, the condition $C_{ij}(t)$ of an individual with alleles a_i and a_j at time *t* is given by $C_{ij}(t) = R_{ij} - \left|G_{ij} - E_t\right|$, where R_{ij} is the resource level of that individual and E_t is the state of the environment at time *t*. The inheritance of alleles is Mendelian in the standard way: an offspring individual inherits one allele from the father and one from the mother, where both alleles of a parent have an equal probability of being transmitted. At the moment of transmission, a mutation may occur with probability μ. If a mutation occurs, a mutational step size δa is drawn from a normal distribution with mean zero and standard deviation σ. Subsequently, the transmitted parental allele a_i is changed to $a_i + \delta a$. Figure 2 was produced by systematically changing μ and σ.

For computational efficiency, we utilise the "infinitely many alleles model" of population genetics (a single locus with many alleles) to implement Mendelian inheritance (Tajima 1996). The "infinitely many sites model" (many diallelic gene loci, each with an infinitesimal effect on the phenotype) is often employed in quantitative genetics, but its use significantly slows down individual-based simulations. However, there are many alternative ways of modelling Mendelian inheritance, and the modeller's choice is always somewhat arbitrary, unless a specific system with well-known genetics is modelled.

3. Replication of Figure 1 in Knell & Parrett 2024

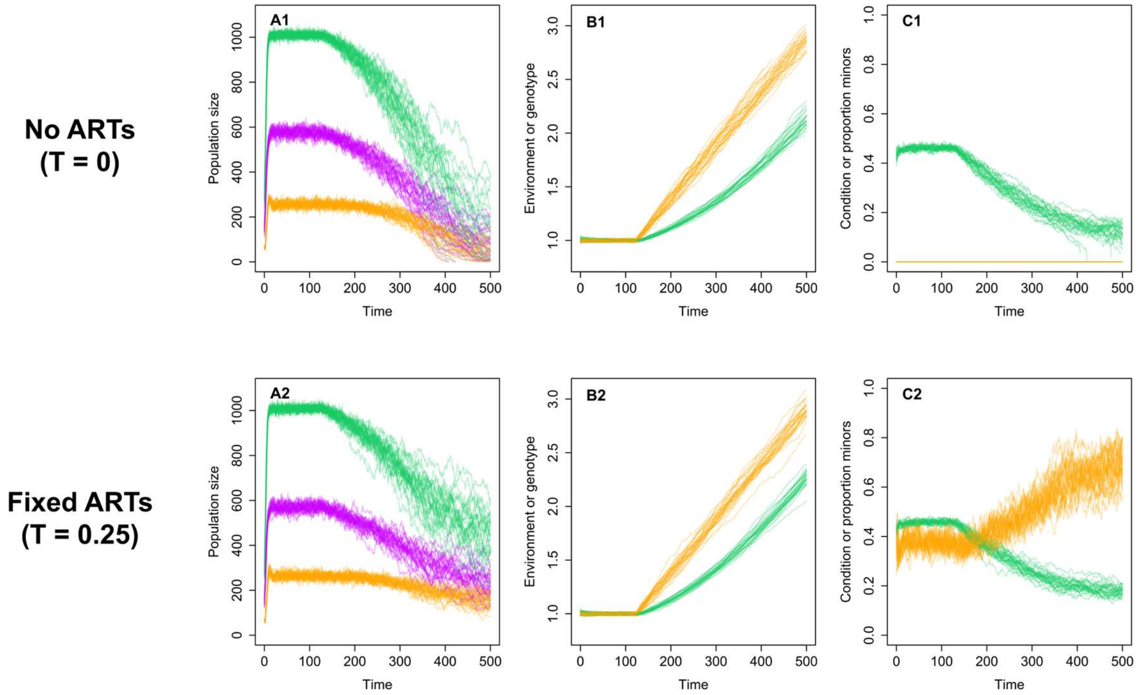

Figure S1: Replication of Figure 1 in K&P 2024. To demonstrate that our re-implementation of the model in K&P 2024 captures the same dynamics as the original model, we have reproduced Figure 1 of K&P 2024, but showing 30 replicate simulations for each scenario (instead of a single, representative one). The top row (panels A1-C1) depicts the dynamics in the absence of ARTs (T=0), the bottom row (panels A2-C2) depicts the dynamics under fixed ARTs (*T*=0.25). **(A) Changes in population size.** Across generations, the number of mature females (purple) and mature males (orange), as well as the total population (green), declines. However, while the population size approaches zero within 500 timesteps in the absence of ARTs, it seems to approach a positive equilibrium level in the presence of fixed ARTs with T=0.25. **(B) Environmental tracking.** After 125 timesteps, the environment E_t (orange) undergoes directional change. The mean "environmental genotype" $\bar{G}$ of the population (green) tracks this environmental change but increasingly falls behind. **(C) Individual condition and proportion of minor males.** As the value of the mean "environmental genotype" falls behind the value of the environment, mean individual condition $\bar{C}$ (green) declines. Consequently, the proportion of males adopting the sneaking strategy (i.e., the proportion of minor males, orange) increases. Panels A1-C1 of our figure correspond to panels A-C of K&P 2024, Figure 1; Panels A2-C2 of our figure correspond to panels G-I of K&P 2024, Figure 1. We did not reproduce panels D-F of K&P 2024, Figure 1, as these depict the dynamics of simultaneous ARTs, which are not the focus of our comment. All parameter values are as in K&P 2024, Figure 1.

4. References

Avise, J.C. (2014). Conceptual Breakthroughs in Evolutionary Genetics. Academic Press.

Tajima, F. (1996). Infinite-allele model and infinite-site model in population genetics. *Journal of Genetics,* 75, 27–31.
